# Advanced Parkinson’s or “complex phase” Parkinson’s disease? Re-evaluation is needed

**DOI:** 10.1007/s00702-017-1799-3

**Published:** 2017-11-07

**Authors:** Nataliya Titova, Pablo Martinez-Martin, Elena Katunina, K. Ray Chaudhuri

**Affiliations:** 1 Department of Neurology, Neurosurgery and Medical Genetics, Federal State Budgetary Educational Institution of Higher Education « N.I. Pirogov Russian National Research Medical University » of the Ministry of Healthcare of the Russian Federation, Moscow, Russia; 2 National Center of Epidemiology and CIBERNED, Carlos III Institute of Health, Madrid, Spain; 3 National Parkinson Foundation International Centre of Excellence, Kings College Hospital and The Maurice Wohl Clinical Neuroscience Institute, Kings College, 5 Cutcombe Road, London, SE59RT UK

**Keywords:** Parkinson’s disease, Advanced, Complex, Nonmotor symptoms, Personalised medicine, Nonmotor subtypes

## Abstract

Holistic management of Parkinson’s disease, now recognised as a combined motor and nonmotor disorder, remains a key unmet need. Such management needs relatively accurate definition of the various stages of Parkinson’s from early untreated to late palliative as each stage calls for personalised therapies. Management also needs to have a robust knowledge of the progression pattern and clinical heterogeneity of the presentation of Parkinson’s which may manifest in a motor dominant or nonmotor dominant manner. The “advanced” stages of Parkinson’s disease qualify for advanced treatments such as with continuous infusion or stereotactic surgery yet the concept of “advanced Parkinson’s disease” (APD) remains controversial in spite of growing knowledge of the natural history of the motor syndrome of PD. Advanced PD is currently largely defined on the basis of consensus opinion and thus with several caveats. Nonmotor aspects of PD may also reflect advancing course of the disorder, so far not reflected in usual scale based assessments which are largely focussed on motor symptoms. In this paper, we discuss the problems with current definitions of “advanced” PD and also propose the term “complex phase” Parkinson’s disease as an alternative which takes into account a multimodal symptoms and biomarker based approach in addition to patient preference.

## Introduction

The concept of “advanced Parkinson’s disease” (APD) remains controversial and unclear in spite of the extensive knowledge of the natural history of the motor syndrome of PD. Largely this relates to the complex pathophysiological process involving dopaminergic and nondopaminergic pathways that underlies Parkinson’s disease (PD) (Uitti et al. 1993; Langston 2006). The concept of advanced PD has been examined in several studies with varying methodologies ranging from retrospective audits to expert opinion and Delphi system based studies usually using disease duration as one of the anchors (Table 1). The terminology has been confusing with varying nomenclature such as late stage or complex stage being used interchangeably while the concept of the palliative stage of PD has also evolved in recent times. The situation is further compounded by emergence of recent knowledge about the key importance of nonmotor symptoms (NMS) of PD, NMS being key determinants of quality of life and not necessarily progressing hand in hand with motor progression of PD (Korczyn 1999; Martinez-Martin et al. 2011; Coelho and Ferreira 2012; Ray Chaudhuri et al. 2013). While advanced motor disease of PD is often associated with a disease duration of 10–15 years and a range of non dopaminergic NMS such as dementia, advanced nonmotor burden of PD may also occur in early motor and even premotor disease (Sommer et al. 2004; Zis et al. 2015; Sveinbjornsdottir 2016; Chahine et al. 2016). While none of the “neuroprotective” strategies attempted in clinical trials have been successful in PD presumably because of the inefficacy of the compounds tested, another cause may be because the therapy is only started at the motor stage of PD, not taking into account the prodromal period when molecular neurodegeneration begins (Korczyn and Hassin-Baer 2015). Studies in experimental models of animals also show a variable association of disease severity with NMS (Titova et al. 2017a). This fact complicates the option of defining “advanced PD”. Furthermore, recent concepts of nonmotor subtypes of PD also indicates that there may be differential rates of progression of PD depending on the natural history of subtypes and hence assigning the concept of APD would need to be individualised as « one size » or definition of APD does not fit all (Korczyn 1999; Sauerbier et al. 2015; Marras and Chaudhuri 2016). The World Health Organisation international classification of disorders (ICD) coding system currently has no definition for APD although many therapies such as levodopa or apomorphine infusion or deep brain stimulation (DBS) are indicated for APD (World Health Organization 2017). In addition, recently, a subgroup of PD has been described, the PD-20 subjects who inspite of 20 years disease duration are relatively independent, show only mild cognitive impairment and some are still working (Hassan et al. 2015). Furthermore, patients often dislike the notion of being labelled with an APD diagnosis and a robust definition of APD would, therefore, be useful from a clinical, prescribing, licensing as well as epidemiological perspective (Table 2). The issue is also relevant for meaningful information sharing. The problems of poor information related to advancing PD and access to information on various “advanced” therapies have been highlighted in a Swedish patient survey of 486 patients where one of the criteria for APD was based on a diagnosis greater than 5 years (Lokk 2011). 1300 subjects responded and 73% were given no information on advanced therapies for PD.

**Table 1:** A summary of studies that have attempted to define advanced PD or late stage PD and provide treatment guidelines

Reference	Type of study	Population base	Outcome
Hely et al. (2005)	Follow up of a cohort recruited to Bromocriptine vs low dose levodopa study	130 patients with 52 surviving at 15 years	At 15 or more years falls, autonomic disturbance, neuropsychiatric symptoms, and dementia cause disability Motor fluctuations and dyskinesias while common are less disabling
Hely et al. (2008)	Follow up of a cohort recruited to Bromocriptine vs low dose levodopa study With 50 age and gender matched controls	30 surviving patients at 20 years FU	83% had dementia along with excessive daytime sleepiness 70%, falls 87%, freezing 81%, symptomatic postural hypotension 48%, urinary incontinence in 71%, hallucinations in 74%
Coelho et al. (2010)	Cross-sectional analysis of an international (Spain-Portugal) out patient cohort	50 PD patients in HY stages 4–5 (late stage) UPDRS, nonmotor fluctuation, cognition and quality of life assessments	Motor and nonmotor (mainly non-levodopa responsive symptoms) were prevalent and the main cause of disability. 50%, however, were considered to be non-demented
Antonini et al. (2015)	Delphi panel—based on consensus	a group of approximately 20 EU neurologists, using 2 rounds of data collection via an online survey and a single in-person meeting	Dementia, hallucinations, psychosis, nonmotor fluctuations, and nighttime sleep disturbances flagged as important potential hallmarks of advanced PD with functional consequences of falls, dependency and risk of pneumonia
Cilia et al. (2015)	Retrospective, cross-sectional study and longitudinal study	Patients with disease duration ≥ 20 years	Older age at onset and longer disease duration independently associated with a higher prevalence of major motor and nonmotor milestones of disease Mortality associated with male gender, older age, dysphagia, orthostatic hypotension, postural instability, fractures and institutionalisation
Odin et al. (2015)	International expert recommendations for the management of PD refractory to oral/transdermal therapies	Collection and consensus of opinions on structured questions from 103 experts from 13 countries overseen by an International Steering Committee (ISC) with 13 movement disorder specialists	Patients requiring levodopa > 5 times daily with severe, troublesome ‘off’ periods (> 1–2 h/day) despite optimal oral/transdermal levodopa or non-levodopa-based therapies considered for advanced therapies even if disease duration is < 4 years Cognitive decline related to nonmotor fluctuations is an indication for device-aided therapies
Luquin et al. (2017)	CEPA Study—a 3-round Delphi study	Including neurologists in Spain and using a Delphi system identification and quantification of clinical variables that characterize patients with APD	Motor syndrome and sleep problems rated as key issues severe dysphagia, recurrent falls, and dementia
Hassan et al. (2015)	International, multicentre National Parkinson’s Foundation Quality Improvement Initiative (NPF-QII) study database used to identify PD-20 subjects	187 PD-20 subjects (55% men) (4% of all NPF-QII participants)	(75%) had 20-25 years of PD duration, longest duration being 49 years. PD-20 subjects reflect an elite group of PD survivors with early onset disease and relatively mild cognitive disability despite long disease duration

**Table 2:** Why we should better define advanced PD

For patients
Provide better biomarker based diagnostic and prognostic evaluation
Improve acceptability of stage (terminology)
For health care professionals:
Improve and consolidate the criteria for diagnosis of APD among clinicians providing standardised criteria
Develop specific pathways which engage movement disorder specialists/“parkinsonologists” so that appropriate guidance for management is initiated early including multidisciplinary services
Provide guidance for appropriate and timely implementation of advanced therapeutic options
Develop appropriate toolkits and scales
For health care providers
Early evaluation of relevant datasets in appropriate group of patients when described as advanced therapies
Facilitate early reimbursement of therapies with reasonable evidence base in appropriate patient groups
Define APD in WHO ICD coding handbook
For developing a true holistic concept
Improve patient care and carer quality of life (affected by the term “advanced” with implications on lifestyle, prognosis as well as social interaction)

From a pathological point of view the concept of APD also remains unclear. Normally the concept of APD is underpinned by a progressive loss of nigrostriatal dopaminergic terminals which eventually disables the buffering capacity to manage fluctuations in striatal dopamine levels (Obeso 2008; Poewe 2010). But it is clear that there is also considerable loss of other neurotransmitter based neurons, sometimes even greater than the dopaminergic loss, leading to the complex syndromic nature of PD (Korczyn 1999; Langston 2006; Titova et al. 2017b). Specifically, the gastrointestinal tract involvement at a cellular and molecular level is now well recognised and gastrointestinal dysfunction common across all stages of PD may lead to erratic/delayed gastric emptying. This may lead to significant changes in plasma levodopa bioavailability related to oral medications (Jellinger 2015; Cerosimo 2012; Poewe 2010; Obeso 2008). Animal model and clinical studies suggest that emergence of motor complications like on–off fluctuations and dyskinesias may therefore be linked at least in part to gastrointestinal dysfunction in addition to central loss of the buffering capacity of the dopamine terminals as the disease progresses (Kordower et al. 2013; Klingelhoefer and Reichmann 2015; Titova et al. 2017b). These patients with PD are likely to exhibit the recently described adrenergic subtype of PD (Jellinger 2015; Titova et al. 2017b). In this paper we discuss these issues and attempt to better conceptualise the definition of APD and also speculate as to whether APD could be better termed as complex phase PD with further relevant subclassifications. We derived the term “complex phase” PD from the concept of “Parkinson complex” originally coined and published by Langston in (2006).

## Barriers for the concept of APD

**Fig. 1:**
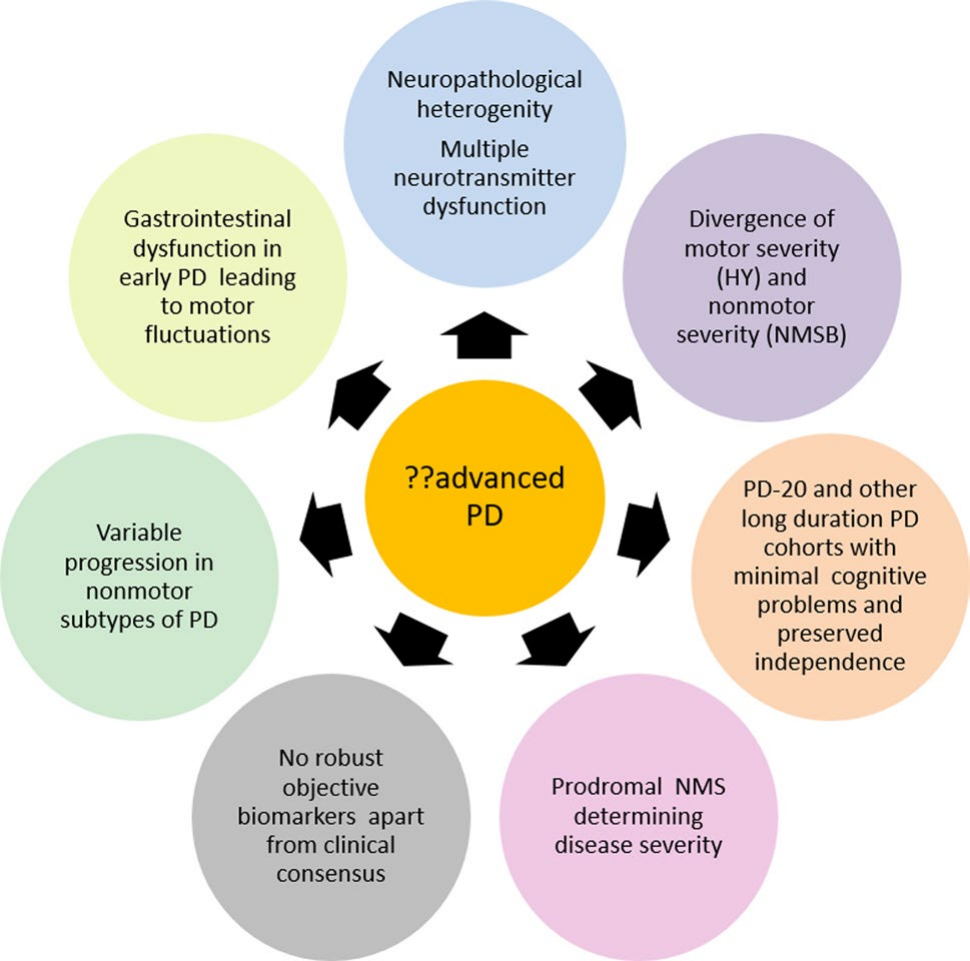
The conceptual and practical problems/barriers to the current attempts at defining advanced PD

Several issues as discussed above complicate the current attempts to define APD and are shown in Fig. 1. These also include recent knowledge that some prodromal symptoms such as cognitive impairment or REM behaviour disorder can dictate the course and severity of manifest PD in addition to the fact there is no specific robust biomarker that can accurately reflect APD.

The need for a robust definition of APD is important for several reasons and these concepts are articulated in Table 2. The issue is first and foremost of great importance to people with PD as the current uncertainties can cause problems with prognostic issues. Critically, it is clear that the prognosis and outcome of the course of PD cannot be generalised and individualisation, an important concept of the personalised medicine strategy is crucial. In addition, the term advanced is poorly accepted by many patients who in spite of a disease duration of 10 years or more may be active, independent as well as cognitively reasonably intact. This suggests disease duration is not a good indicator of severity of PD as will be discussed later. A robust and clinically relevant definition of APD is first and foremost important for patients (Table 2). Clinical experience suggests that many patients resent being called “advanced” particularly if they are functionally active, able to be still working and socialise although with some limitations. Such case examples have been recorded in clinical experience. “Advanced” terminology also has considerable impact on working contracts, employer’s attitudes as well as inter-personal relationships. An alternative term that better defines an objective and dynamic way that defines “APD” is therefore important for patients.

Other factors include implications for health care professionals and commissioners/health care providers who need a standardised criterion which can influence pathways of treatment and in particular advanced therapies. The issue is of great importance for health care providers as well as commissioners who provide reimbursement for treatment based on a diagnosis of “APD” in some cases. A consistent definition would reduce considerable variability in treatment as well as ensuring that patients are not denied advanced therapies when in spite of relevant symptoms they may not satisfy current definitions of APD. The rationale for development of evidence based medicine is based on this concept.

## Problems of defining advanced PD with disease duration

Traditionally “long” disease duration has been the standard method and anchor by which APD has been defined although in most cases PD duration is really a secondary marker. Publications attempting to define APD thus comprises of alternative concepts of definition of APD, for instance, using a consensus versus those where a patients symptom-state at 15, 20, or more years of duration were considered (Table 1). The latter concept therefore, was focussed on the predominant clinical manifestations at that time and described by staff of tertiary specialized centres and not gleaned from true population based data.

The length of the disease duration for PD to be considered as APD has been based on the duration of the “honeymoon” period or “stable” PD and emergence of motor complications (Sveinbjornsdottir 2016; Titova et al. 2017c). This length of disease duration is variable and in some studies a disease duration of 5 years has considered to be a short disease duration while in some studies a disease duration of 5–6 years is considered advanced disease (Simuni et al. 2013; Pistacchi et al. 2017; Politis et al. 2010; Lokk 2011). Signs that have been thought to define APD have also been variable and a recent three round Delphi consensus-based criteria has been proposed from the Spanish CEPA study (Luquin et al. 2017). In this consensus, the disease duration anchor of 10 years (median) was one of the markers for APD and in addition, definitive symptoms of APD included disability requiring help for the activities of daily living and/or severe dysphagia, recurrent falls, and dementia. However, several studies have examined PD patient cohorts with a disease duration of 20 years or more and most recently a study reported that this PD-20 cohort do not satisfy the criteria for APD as defined by the consensus criteria (Hassan et al. 2015; Cilia et al. 2015). Disease duration alone therefore cannot truly map whether PD is advanced or not and certainly there is no “inevitability” about dementia at 20 years PD as has been suggested from the follow up report of the Sydney multicentre study (Hely et al. 2008). In addition, subtle cognitive impairment has now been described in the prodromal stage of PD even before the motor symptoms are evident (Weintraub et al. 2015; Chahine et al. 2016). The uncertainty about definition of APD is also reflected in the current International Classification of Disorders Classification of Parkinson’s Disease where currently, there is no subcode specifically for APD [World Health Organization (2017) G20: Parkinson disease].

As discussed before, several workers have addressed the concept of APD, some using a longer disease duration (10–20 years) as anchor, but as a secondary marker (Hely et al. 2005, 2008; Cilia et al. 2015). Symptoms included are a range of motor and nonmotor symptoms while others have relied on expert opinion based or Delhi type consensus.

In a meta-analysis performed by Chen et al. (2015) the duration of illness primarily correlated with the frequency of constipation and excessive daytime sleepiness. However, symptoms such as apathy, attention/memory, psychiatric symptoms did not correlate with disease duration. In particular, psychiatric NMS, mainly anxiety and depression, were reported by 61.1% of patients at Hoehn and Yahr stage 1 illustrating the problems of defining disease severity of PD with disease duration (Chen et al. 2015). This observation as well as the variability of the outcome in studies as shown in Table 1 underpins the need to develop a multimodal strategy for defining APD and not just rely on disease duration, and symptom occurrence.

## Are there biomarkers for advanced PD?

Biomarkers are important to support the concept of APD and can be classified to (Sharma et al. 2013; Titova et al. 2017c).

- In vivo (neuroimaging, neurophysiology, polysomnography)
- In vitro (biochemical/genetics from tissue samples)
- Pathological (α-synuclein)
- Neuroimaging: dopamine transporter loss (Datscan putamen binding ratios), iron accumulation (cranial ultrasound)
- Neurobehavioral (depression, fatigue, cognition)
- Other clinical symptoms

Lerche et al. (2016) has proposed a set of clinical and objective biomarkers to better define stages in longitudinal studies of PD. Nine modalities ranging from motor, neuropsychiatric, other nonmotor clinical symptoms to imaging and blood based and other biomarkers such as skin biopsy have been proposed. Specifically, autonomic, gastrointestinal, sleep and sensory nonmotor measurements are listed. A recent study has highlighted the possible role of skin biopsy and detection of abnormal alpha synuclein in cases with rapid eye movement behaviour disorder (Doppler et al. 2017). These modalities may be useful for research based studies, but for real life clinical assessment of APD a more pragmatic and simpler paradigm is required. Kruger et al. (2017) have proposed the concept of “deep phenotyping” which includes device based assessments (such as accelerometers or smartphones), molecular and genetic biomarkers as well as clinical subtyping of patients to better define the stages of PD leading to personalised precision medicine delivery. However, the role of biomarkers to define APD is far from clear. A battery of biomarkers is clearly required and recent evidence from a 2-year follow up study of de novo PD suggests that single and cognitive biomarkers are not be adequate to map progression of PD (Mollenhauer et al. 2016). Thirty possible biomarkers were examined in this study and the authors conclude that a multimodal approach, with clinical, biochemical, and laboratory based biomarkers, is needed (Mollenhauer et al. 2016). It must be recognised, however, that at present modalities such as transcranial ultrasound or skin biopsy remains investigational as biomarkers. Dopamine transporter imaging while useful as a surrogate marker for dopaminergic deficiency remains impractical for routine use because of cost.

A important new advance is the role of technology-based devices which can be classified to wearable, non wearable, and hybrid devices (Godinho et al. 2016; Bhidayasiri and Martinez-Martin 2017). Early findings from studies assessing the role of applications of these technologies suggest that these technologies may support and effectively track the clinical assessment of motor features of PD, certain NMS such as sleep, as well as gait, mobility, risk of falls daily living transitions, and physical activities. Several of these symptoms are included in the Delphi consensus based definition of APD (Antonini et al. 2015) and as such these devices may serve as possible biomarkers and, importantly, provide objective data supporting the diagnosis of the APD state.

## How can the concept of APD be improved? Proposal to change terminology to complex PD

It is clear that our current concept APD is plagued by several problems including unclear transitional milestones when stable PD becomes unstable PD or early PD becomes APD. The syndromic nature of PD, multi neurotransmitter involvement, motor and nonmotor subtypes of PD, variable and unclear progression pattern of motor and nonmotor symptoms and recent mapping of several NMS marking prodromal PD make a clear definition of APD inaccurate as largely the concept is underpinned by disease duration. Feedback from patient groups as well as clinical experience suggests that some patients also are troubled by the term APD when they may still be relatively independent. Our proposal is that a better alternative terminology to use would be complex phase PD. Complex phase PD would encompass the basic concept of the complex origins of PD as well as the heterogeneity of its clinical presentation. Factors and mechanisms that may support the concept of using the term complex phase PD instead of APD are shown in Table 3.

**Table 3:** Enablers and factors that may suggest the term complex phase PD is a better term to use to denote advanced Parkinson’s disease (PD)

Patient preference
Complexity of mechanisms that lead to the clinical expression of motor and nonmotor subtypes of PD
The need for multimodal and not single biomarkers
The need to take into account various strands of personalised medicine
The fact that advanced PD implies duration of disease while complex refers to the conditional stage and incorporates the concept that early PD can also be complex

Currently there is no consensus or list of factors that may constitute the definition of complex phase PD. Our proposal is that several factors as shown in Table 4 which takes into account the list of enablers shown in Table 3 could be used to define complex phase PD. Clinically this will include gradation of PD using a cumulative grading system of motor and nonmotor symptoms. Such scales and its burden grading has been described and the most pragmatic and easy to use system would be the combination of the Hoehn and Yahr staging (HY, motor) with nonmotor burden grading either using the NMS questionnaire or NMS scale (Sauerbier et al. 2015). NMSQuest grading is currently the only validated NMS grading using a patient related outcome measure. Alternative grading systems could also be used such as combining HY staging with the part 1 of the movement disorder society unified Parkinson’s disease rating scale (MDS-UPDRS). This strategy moves away from specific motor and NMS which have been cited in the consensus criteria for APD as such symptoms can also occur in “early” PD. Examples would be, the occurrence of cognitive impairments in prodromal PD as well as a considerable burden of NMS even in drug naïve PD (Chahine et al. 2016; Zis et al. 2015). However, such a strategy alone would not be sufficient. In addition, the complexity of PD and need for advanced therapies would also be underpinned by failure of “conventional therapies, usually oral and transdermal dopamine replacement therapies as well as emergence of troublesome motor and nonmotor fluctuations to levodopa therapy. The clinical grading would need to be supported by selected biomarkers. As discussed above currently, the role of these biomarkers are unclear but based on clinical experience low putamen binding ratios on Datscan and or low caudate and putamen binding rations can be used to mark complex PD. In future sophisticated MRI imaging techniques such as T2-weighted fluid-attenuated inversion recovery sequences for detection of nigral or basal forebrain degeneration, diffusion tensor imaging for subcortical white matter degeneration as well as voxel-based morphometry may also emerge as useful surrogate biomarkers. The role of device based assessments (accelerometry based tracking of bradykinesia as well as dyskinesia and some nonmotor symptoms) is being investigated and such tools are already in routine clinical use in many countries. Finally, the role of personalised medicine for PD has merged as a specific and not simply a genomic strategy (Titova and Chaudhuri 2017). Complexity of PD should therefore, also take into consideration the factors that complete the “circle” of personalised medicine and specifically assessment of personality traits. A summary of proposed factors that could constitute the concept of complex phase PD is shown in Table 4.

**Table 4:** Factors proposed to define complex phase PD

Combination of motor and NMS grading using a combined grading system (HY + NMSSB or HY + NMSQB)
Falling or failing response to oral/transdermal DRT therapy with emergence of motor and nonmotor fluctuations
Datscan showing low (dependent on local laboratory estimates) putamen or combined putamen and caudate binding ratios
Accelerometry based dataset indication and supporting clinical impression of severe bradykinesia or troublesome dyskinesia at home. (Additionally some nonmotor data may also become available such as an indication of sleep function)
Presence of personality trait based factors necessitating the delivery personalised medicine

*HY* Hoehn and Yahr, *NMSSB* nonmotor symptoms scale derived burden, *NMSQB* nonmotor symptoms questionnaire based burden

It is also conceivable that in future one would be able to define patients who may be “at risk” of developing complex PD perhaps earlier than other patients with PD. Such patients may be the ones with severe and troublesome RBD during the prodromal stage who are more likely to have cognitive dysfunction, dysautonomia as well as falls (Postuma et al. 2015). Olfactory dysfunction may further enhance the development of dementia (Takeda et al. 2014). In addition, we would envisage that complex phase PD could also be stratified to subgroups based on clinical neurotransmitter dysfunction driven subtypes, complex early and complex advanced stages, biomarkers, response to current therapies as measured by the APD toolkit.

## Scales and toolkit for complex phase PD

Attempts to develop a validated toolkit for better definition of APD have been initiated. The toolkit is based on measurement of inadequate control with oral anti-PD medications as well as a frequency ad severity graded assessment of a collection of motor, nonmotor symptoms (nonmotor fluctuations, psychosis and impulse control disorders) and symptoms of functional impact (frequency of falls and increased dependency) (Antonini et al. 2017). Validation of this toolkit is awaited. Whether such a toolkit will accurately reflect our concept of complex phase PD also remains to be tested.

## Conclusion

The term APD remains poorly defined and a robust definition remains an unmet need inspite of consensus opinion. Patient acceptance of the definition of advanced PD is poor and clinically the concept needs to include our growing knowledge of the heterogeneity and syndromic nature of PD underpinned by motor and nonmotor subtypes as well as the variable progression pattern of the motor and nonmotor aspects of the disorder. The issue is of great importance as provision for advanced therapies for PD and personalised medicine strategies are based on the current definition of APD. We propose an alternative approach to the concept of defining APD which may be better termed complex phase PD taking into account a clinical and biomarker based multimodal approach. This concept needs to be examined in real life studies and may better define the prospect of personalised medicine delivery as well as prognostic aspects of this complex disorder.
